# Gel Point Determination in Resin Transfer Molding Process with Fiber Bragg Grating Inscribed in Side-Hole Elliptical Core Optical Fiber

**DOI:** 10.3390/ma15186497

**Published:** 2022-09-19

**Authors:** Karol Wachtarczyk, Marcel Bender, Ewald Fauster, Ralf Schledjewski, Paweł Gąsior, Jerzy Kaleta

**Affiliations:** 1 Department of Mechanical Engineering, Wrocław University of Science and Technology, Smoluchowskiego 25, 50-370 Wrocław, Poland; 2 Processing of Composites Group, Montanuniversität Leoben, Otto Glöckel-Straße 2/III, 8700 Leoben, Austria

**Keywords:** fiber optic sensors, Fiber Bragg Grating, high birefringent fibers, side-hole optical fiber, resin transfer molding, cure monitoring, gel point estimation, residual stress

## Abstract

Material as well as process variations in the composites industry are reasons to develop methods for in-line monitoring, which would increase reproducibility of the manufacturing process and the final composite products. Fiber Bragg Gratings (FBGs) have shown to be useful for monitoring liquid-composite molding processes, e.g., in terms of online gel point detection. Existing works however, focus on in-plane strain measurements while out-of-plane residual strain prevails. In order to measure out-of-plane strain, FBG inscribed in highly birefringent fiber (HB FBG) can be used. The purpose of this research is the cure stage detection with (a) FBG inscribed in single mode and (b) FBG inscribed in highly-birefringent side-hole fiber in comparison to the reference gel point detected with an in-mold DC sensor. Results reveal that the curing process is better traceable with HB FBG than with regular FBG. Thus, the use of HB FBG can be a good method for the gel point estimation in the RTM process.

## 1. Introduction

Differences in batch-to-batch properties of textiles and resins lead to variations in the manufacturing process conditions. The effect of these changes can cause defects in the final part (dry spots, undercuring) [1]. To address this problem, online monitoring of the composite manufacturing process is applied [2,3]. In the case of the RTM process, it is done with in-mold sensors, but also with embedded ones [1,2,4,5,6]. Among the embedded ones, fiber optic sensors (FOS) have received a lot of attention [1,2,4,5,6,7,8].

FOSs are used predominantly for structural health monitoring (SHM) of composite parts [9,10,11,12,13,14]; their small size, immunity to the electromagnetic field, and multiplexing abilities make them perfect for this solution. However, because FOS are embedded on the stage of composite production, it is a great opportunity to use them also as sensors for the manufacturing process.

One of the most popular FOS is Fiber Bragg Grating (FBG). A basic FBG is a few-millimeters-long section of a fiber, with in-written periodic modulation of the refractive index [15]. The distance between these modulations (grating period) and the effective refractive index of the fiber’s core determine a wavelength of light reflected by the FBG (so-called Bragg wavelength). Axial strain applied to the optical fiber causes changes in grating’s period, which can be measured as variations in the wavelength of light reflected from FBG [16].

FBG sensors are used in thermosetting composites to detect gel points and measure the overall residual strain accumulated in the composite [4,5,6,17,18,19,20]. A gel point detection with FBG sensors is based on the search for small changes in strain trends recorded by FBGs [4,6,17]. In these papers, it is said that gelation causes the resin to harden enough to transfer stresses to the sensor, which allows strain buildup measurement with sensors; these trend variations are small, typically on the order of 100 με after the entire curing process (chemical residual deformations) [4,6,18].

Such small strain amplitudes require methods to increase the precision of the gel point determination. In paper [4], the reproducibility of process monitoring was improved by placing the sensor in resin pockets or small tubes so that the measurement could be performed under controlled conditions. Another work [17] describes the detection of subsequent production stages by investigating neat resin’s curing. In the sample an FBG and thermocouple were placed, and additional thermocouple was placed outside of the sample. Heat transfer and strain results allowed the description of the following steps of the process. Comparison of the performance of FBG sensors inscribed in 150 µm and 80 µm single-mode fibers for vacuum infusion process monitoring was shown [19] and both of the sensors showed comparable performance.

However, in all the cases presented, the main detection method with FBGs is the measurement of deformations, which occur in the plane of the composite, while a residual in-plane strain is often much smaller than in the out-of-plane direction [21,22,23]. One way to measure out-of-plane strain is to place a Bragg grating perpendicularly to the composite plane [21]. In this way, it is possible to directly measure out-of-plane strain, but the method is not suitable for closed-mold methods and is limited to thick laminates.

Another option to measure out-of-plane strain is to use FBG inscribed in highly-birefringent fibers (HB FBG). A reflection spectrum of HB FBG consists of two separate peaks, whose separation is related to the transverse stress state in the optical fiber core; this means, if the optical fiber is loaded with an external transversal load, the separation between the peaks will change [24,25,26]. In the case of HB FBG embedded into the composite, the out-of-plane shrinking will cause a perpendicular load on the optical fiber, thus changing the separation of the peaks.

This approach has already been presented in several publications [22,24,27,28]. There, the authors demonstrated the possibility of measuring deformations during [22,27] and after [24] the entire manufacturing process (residual ones). In [27], the measurement of out-of-plane strain is described in a unidirectional glass-epoxy composite and the results are correlated with DSC data to obtain the degree of cure. The paper [24] showed that HB sensors can measure the total transversal residual strain after curing and cooling the composite after the autoclave manufacturing process, while the work [22] presented the use of multiple HB FBGs for transversal strain measurement and multiple SM FBGs for axial strain measurement during the manufacturing and after the process of an aircraft tail cone.

Previous works (Table 1) focused on the process monitoring performed either with FBG inscribed in single-mode fibers, or in highly-birefringent fiber. In the paper [22] monitoring with both types of FBG sensors was shown, but it was done for the autoclave process, no cases of comparison of the performance of HB FBG with SMF HB for closed mold processes was presented before. Additionally, in this paper FBG sensor is inscribed in novel HB fiber (side-hole fiber with an elliptical core), which has increased sensitivity to transversal in comparison to bow-tie fibers and detection in this direction is almost insensitive to temperature.

Because of the identified knowledge gap, this paper will focus on the comparison of resin transfer molding process monitoring with FBG sensors inscribed in single-mode fibers with an FBG inscribed in highly-birefringent optical fiber. To address this issue, the following tasks were performed:

1. Detection of gel point according to a reference method (in-mold DC and temperature sensor).
2. Monitoring of curing process with regular FBG.
3. Monitoring of curing process with FBG inscribed in highly-birefringent side-hole fiber.

## 2. HB FBG Sensors

An FBG inscribed on single-mode fiber reflects a narrow band of light passed through the optical fiber. The wavelength of reflected light is given by:

(1) $$\lambda_{B}=2\Lambda n_{eff},$$

where $\lambda_{B}$ denotes the reflected wavelength, Λ terms the period of FBG and $n_{eff}$ refers to the effective refractive index of the fiber core. If the FBG is subjected to axial deformation or temperature, the Bragg wavelength changes [16]. In the case of highly birefringent fiber, stresses induced in the fiber core during the fiber’s manufacturing causes that $n_{eff}\;$ differs for orthogonal axes. In this case, the reflection spectrum of a single FBG contains two peaks, given by the equations:

(2) $$\{\begin{array}{c} \lambda_{B_{x}}=2n_{eff_{x}}\Lambda ,\\ \lambda_{B_{y}}=2n_{eff_{y}}\Lambda . \end{array}$$

A peak with a lower wavelength corresponds to the fast axis ($\lambda_{f}$) of the fiber and the one with a higher wavelength to the slow axis ($\lambda_{s}$). The distance between peaks depends on the stress state in the cross section of the fiber core. If an external transverse load is applied to the fiber, the stress state in the core changes, causing variation of this stress state and changes in peak separation. On the other hand, if load is applied along an HB FBG, both peaks move parallel, because of grating pitch changes.

**Figure 1:**
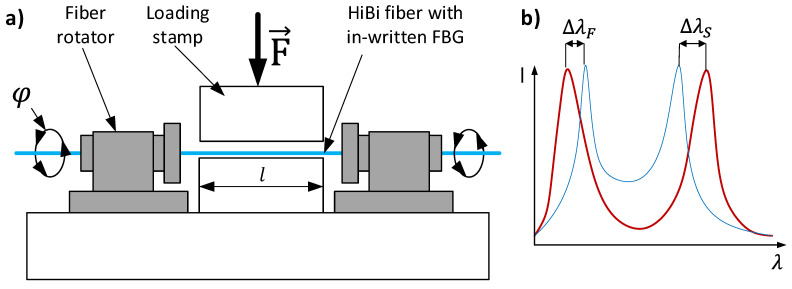
Testing of an HB FBG sensor; (**a**) a scheme of testing, (**b**) variation in Bragg wavelengths due to external force.

The presence of polarization axes causes that the response for the external load applied to the transversal part of the fiber is dependent on the angular orientation of a fiber. To test this dependency, fiber was tested under the external lateral force applied on various angles. The procedure is carried out in a stand as shown in Figure 1.

Fiber rotators are used to change the angular orientation of a fiber (φ). When fiber is positioned, loading stamp compresses an optical fiber with in written FBG. For each tested angle, sensitivity of both Bragg peaks and separation dependency to the external force is measured. Sensitivity for an external force can be described as:

(3) $$s_{s,f}\left(\varphi ,F\right)=\frac{\Delta \lambda_{s,f}}{F/l},$$

where $\Delta \lambda_{s,f}$—Bragg wavelength shift, F—force applied to the tested fiber l—length of a fiber under the compressive stamp. In the same way, the so-called effective sensitivity ($s_{eff}$) can be calculated. In this case, a peak separation variation is used instead of Bragg wavelength shift.

**Figure 2:**
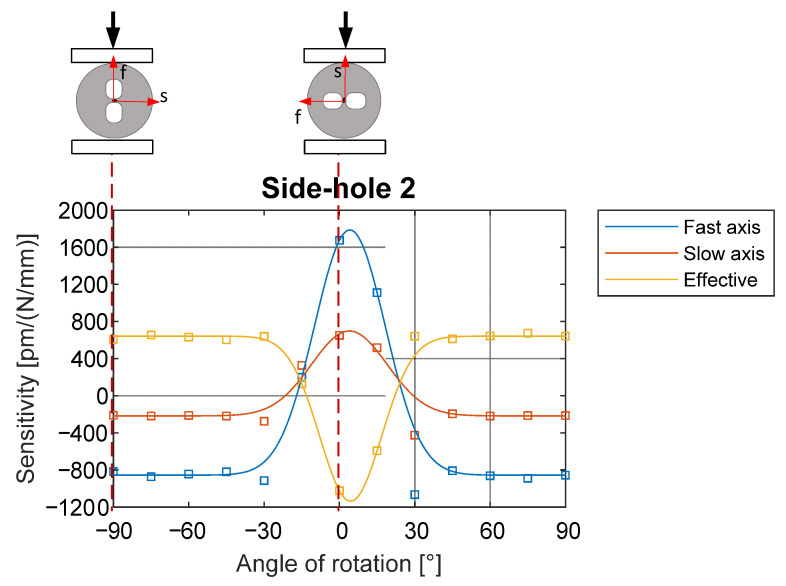
Sensitivity of peaks in the side-hole fiber under the force-loading conditions. Based on [29].

In this paper, the highly-birefringent fiber that will be used to measure strain, is a side-hole optical fiber. Figure 2 shows the variation of the FBG peaks under the transversal force applied to the fiber on different angles.

If force is applied along the slow axis of a fiber (angles −30°–+30°) the effective sensitivity to the load is negative, so the separation of peaks decreases; this results in the highest absolute value of 1150 pm/(N/mm). Meanwhile, when fiber is compressed along the fast axis or in the surrounding range (angles −90°–−30° and +30°–+90°) compressive force increases the peak separation. In this case sensitivity is around 650 pm/(N/mm) [29].

**Figure 3:**
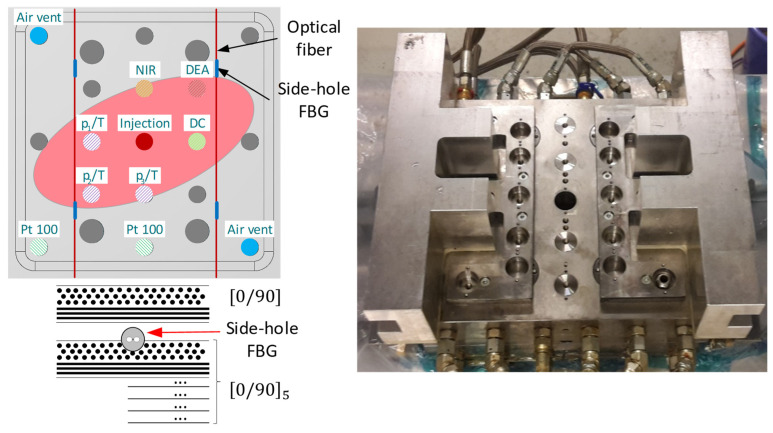
Top view on the upper mold half, showing available sensor positions, sensor fixations and the central injection point (**right**). Schematic view of the upper mold half, showing sensor types and positions used for the measurements as well as the expected flow front orientation (**left**).

In the work at hand, to have the highest sensitivity to the shrinkage in the out-of-plane direction, HB FBG was used in an orientation corresponding to the 0° angle loading case (slow axis is perpendicular to the plane of the composite). Location and orientation of FBG is shown in Figure 3 (left).

Moreover, the stacking sequence of textiles and composite stiffness influence strain transfer to the fiber (true strain measurement). Because of this reason, sensitivity of HB FBG to the external strain was measured experimentally in mechanical testing of manufactured structure. Testing of the samples was presented in [29]. In these conditions, mechanical calibration has shown that if load is applied in the direction perpendicular to the optical fiber, peak separation changes by 0.120 pm/με [29].

Sensitivity of the use of regular FBG sensors versus HB FBG sensors can be described. In the case FBG sensors, the peak detection is performed with a resolution of 1 pm. FBG sensors depending on the optical fiber exhibit various sensitivities to strain and temperature. AN FBG inscribed in single-mode fiber has sensitivity of 0.84 με/pm and 0.1 °C/pm [1] for strain and temperature respectively. FBG sensors inscribed in HB side-hole fiber are used for transversal strain sensing. In this case sensitivity is 0.12 με/pm [29], so the sensitivity to transversal strain is 7 times lower than the sensitivity for the axial strain. However, peak separation is much less influenced by temperature. Peak separation changes with temperature with a sensitivity of 5.88 °C/pm [29]. Active control temperature system ensures temperature variations will not exceed a few degrees, so peak separation changes due to temperature variations is negligible.

## 3. Manufacturing Process

The RTM process was carried out in a closed steel mold, with the cavity having a geometry of a flat, square plate with dimensions of 270 mm × 270 mm and a thickness of 4 mm. By using a single heating/cooling unit and connecting both mold halves in parallel, a homogeneous and constant mold temperature was achieved. Since both mold halves are almost identical in size and geometry and the cooling/heating channels are positioned the same, a constant temperature of 100 ± 0.5 °C over the cavity surface can be guaranteed throughout the entire manufacturing process. After reaching the processing temperature, a further 10-min waiting period ensured that the entire mold reached a stable temperature level. Then six 270 mm × 270 mm layers of a bonded carbon fibre non-crimp fabric (NCF) (Saertex X-C-PB-555), cut with a ZÜND CNC cutter, were placed in the cavity of the same size. After closing the cavity, a Tartler injection unit (type Nodopur VS-2K) was used to ensure (a) constant mixing quality of the two matrix components, (b) a constant injection pressure during the injection period and (c) a fully saturated textile. Furthermore, a so-called flushing phase was added after complete mold filling; this flushing phase allows the removal of the resin at the end of the cavity, which captures the air bubbles trapped in the textile during injection and pushes them out of two tubes at the back of the cavity. Once no more air bubbles are purged from the cavity, the outlets are closed.

The fiber-volume-fraction (*FVF*) of 0.47 of this specimen was calculated according to Equation (4) from the volumes of fibre and matrix; these in turn can be calculated, according to Equations (5) and (6), from the dry ($m_{dry})$ and saturated weight $\left(m_{saturated}\right)$ of the textile, as well as from fiber ($\rho_{F})$ and matrix density ($\rho_{M})$, as specified in the respective technical data sheet of the manufacturer.

(4) $$FVF=\frac{V_{F}}{\left(V_{F}+V_{M}\right)}$$

(5) $$V_{F}=\frac{m_{dry}}{\rho_{F}}$$

(6) $$V_{M}=\frac{m_{saturated}-m_{dry}}{\rho_{M}}$$

A compaction to this *FVF* proved non-critical for FBG-sensors during previous compression tests for all tested lay-up options. When more layers of NCF were compressed to 4 mm, optical fibers were sometimes broke or FBG spectra were significantly distorted.

To monitor the process, optical fiber with FBGs was placed between the fifth and sixth layer to ensure maximum bending sensitivity during later mechanical tests. Bragg gratings were placed 170 mm apart, at the corners of the square (each 50 mm from the edge of the plate). The placement of FBG sensors is shown in Figure 3. One side of the optical fiber was glued to the fabric while the second was loaded with a hanging weight to cause pretension to the FBG. The interrogation was performed with HBM SI405 (Darmstadt, Germany) with Bragg wavelength determination with 1 pm resolution. In case of regular FBG sensors, a 1 Hz sampling rate with peak tracking (automatically) was used. To increase robustness in the case of HB sensors, for the first 30 min optical spectra were collected every 10 s and peak detection was performed with the cross-correlation method [30]. Besides, an automatic peak tracking at 1 Hz was used. Temperature compensation was based on the in-mold temperature sensors. FBG sensors used for the monitoring were commercial fiber optics sensors with polyimide coatings (Sylex FFA-01, Wetherill Park, Australia) and side-hole highly-birefringent fibers.

The roughness of both fibers was inspected with SEM. Both surfaces of optical fibers were smooth without observable structural roughness. In the case of side-hole fiber, the optical fiber surface is smooth and without any flaws. The polyimide coating of the single mode fiber is smooth, but have single surface defects with sizes in the 5–10 µm range. In this case it can be concluded that the roughness of the fiber surface should not influence the comparison of the performance of FBG sensors inscribed in both fibers.

When preform with FBG sensors was inserted, the mold was closed and locked with a force of 40 kN, ensuring a sealed cavity during the injection process. A mass fraction of 100:32 of preheated (50 °C) Epinal 77.55-A1 resin and Epinal 77.55-B1 hardener was injected into the cavity at a constant mass flow rate of 0.3 kg/min until complete filling. The injection was then continued for 10 more seconds, until there were almost no air bubbles left in the transparent pipes leaving the air vents, finishing the so-called flushing phase. Mold temperature was kept at a constant 100 °C with a mold heating system. Due to the good heating situation, both the injection and curing phase can be seen as isothermal.

The plate mold used for these measurements is integrated into a Langzauner LZT-OK-80-SO mold carrier (Lambrechten, Austria). The plate mold itself is manufactured to monitor the RTM process during closing, injection and curing. For this, the top half of the mold allows for up to 21 different sensors to be placed in a grid, depending on the type of measurement and material used. Figure 3 shows a top view of the mold, available sensor positions in the grid (dark grey) and the used sensor types and their respective placement.

The main focus on the placement of the built-in sensor types was to detect the flow front arrival and, thus, flow front propagation. Three combined pressure/temperature sensors (p/T), a combined DC/temperature (type K thermocouple) sensor, detecting changes in resistance from dry to saturated preform state, and two temperature sensors (Pt100) were used. Besides flow front arrival, the DC-sensor (Synthesites optimold) can also detect chances in the matrix material e.g., the degree of cure. The additionally installed Near Infra-Red (NIR) sensor detects changes in the molecular composition of the resin and, just as the DC-sensor, detects flow front arrival and degree of cure. The DEA-sensor (INASCO cure monitor) can detect a wide range of process parameters such as gel point, reactivity and diffusion properties through the functional principal consistent with that of an impedance measurement. The combination of the measurement data from the built-in sensors can then be compared to the data of the FBG-sensors in the textile. Through this, specific changes in FBG-sensor data can be attributed to important process steps, e.g., flow front arrival, gelation, part separation from the mold and a built-up of internal stress due to matrix shrinkage.

## 4. Results

### 4.1. Process Monitoring with Regular In-Mold Sensors

**Figure 4:**
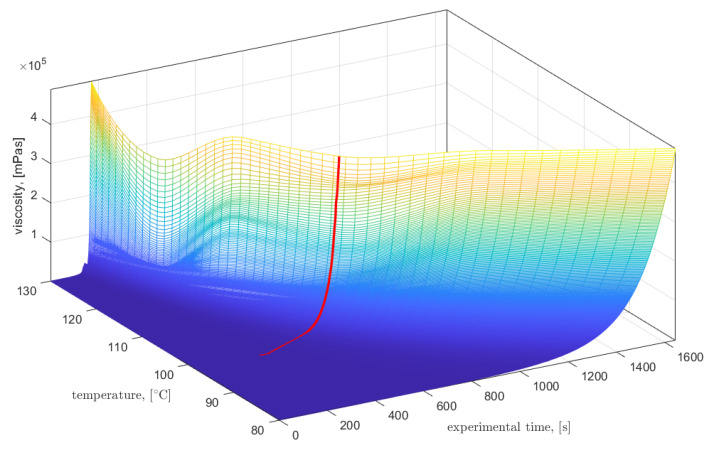
Viscosity field of the resin system (Epinal 77.55-A1/Epinal 77.55-B1) depending on time and temperature, as found from offline characterization, with an overlay of the temperature-time path, as measured with the in-mold DC sensor.

The gel point of the resin system was estimated by processing the temperature and electric resistance data acquired with the in-mold, combined DC/temperature sensor according to a method reported in [31]. In a series of six isothermal runs between 80 °C and 130 °C (constant steps of 10 °C), the viscosity of the thermoset resin system was experimentally characterized at first. For this purpose, an Anton Paar MCR302 rheometer device (Graz, Austria) was used in rotational mode with a shear rate of 10 1/s and a shear gap of 1 mm. The resulting data was linearly interpolated to provide a viscosity field over time and temperature as visualized as a surface plot in Figure 4. The temperature data acquired during the RTM experiment with the in-mold, combined DC/temperature sensor was then interpolated into the viscosity field, resulting in a time-dependent resin viscosity path as shown by the overlay in Figure 4.

**Figure 5:**
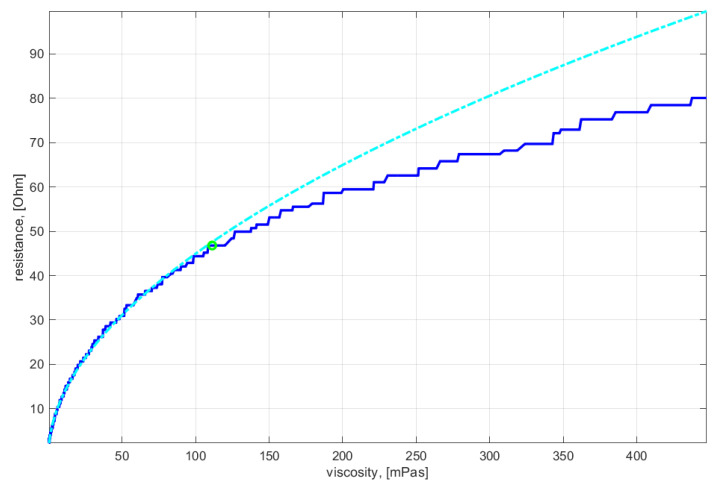
Correlation of electric resistance data with thermoset resin viscosity (dark blue), together with the model curve (light blue) corresponding to the point of maximum ‘goodness-of-fit’.

This allows for correlating the resin viscosity η extracted from the data field with the corresponding electric resistance data R. According to [32], the following model is applicable for the thermoset resin system under consideration in the pre-gelation state:

(7) $$R=\frac{C}{\eta^{k_{v}}},$$

with the model parameters C and $k_{v}$, respectively. A step-wise model fitting approach was used to determine a ‘goodness-of-fit’ measure of the model over the resistance-viscosity data. The maximum of this ‘goodness-of-fit’ measure indicates the gel point, as the model cannot be approximated with higher accuracy beyond this point. Figure 5 shows the characteristics of electric resistance over resin viscosity together with the location of the maximum ‘goodness-of-fit’ measure and the corresponding model characteristics. Finally, back-substitution into the data and subtraction of the injection duration yields an estimate of the gel time.

### 4.2. Regular FBG Sensors

**Figure 6:**
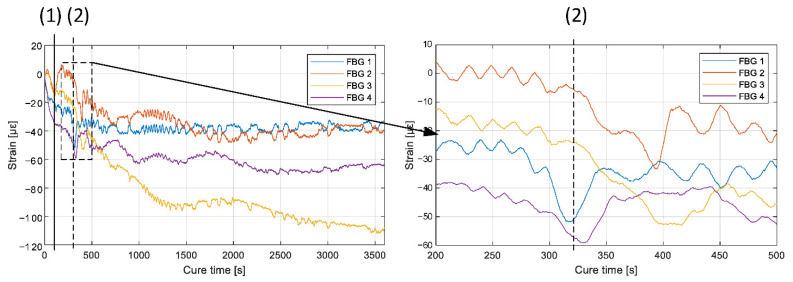
Strain evolution during the RTM process, measured with single-mode FBG sensors; (1)—inlet hose clamping, (2)—range of gelation time estimated from DC sensor data.

In the first test, four commercial FBG sensors inscribed in single-mode were attached to the preform and inserted into the mold. Results of the strain data calculated for four sensors in a single plate during the curing are shown in Figure 6.

In point (1), the inlet hose was clamped, resulting in a sudden change in strain measured with FBG. On the basis of the DC sensor data, gelation time was estimated to be around 322 s. In this time on the plot, significant changes can be observed in the calculated strain data; they are not entirely synchronized due to slow flow rate, which results in differences in the time when resin arrived at each FBG sensor. After the gel point, the chemical reaction proceeds and a slow trend of compressive strain build up is visible. After 2400 s strain stabilized, which could be attributed to the full-cure time.

After cooling down of the plate neither spectrum degradation, nor induced birefringence of FBGs was observed. The final residual strain was −1100 με.

### 4.3. HB FBG Sensors

**Figure 7:**
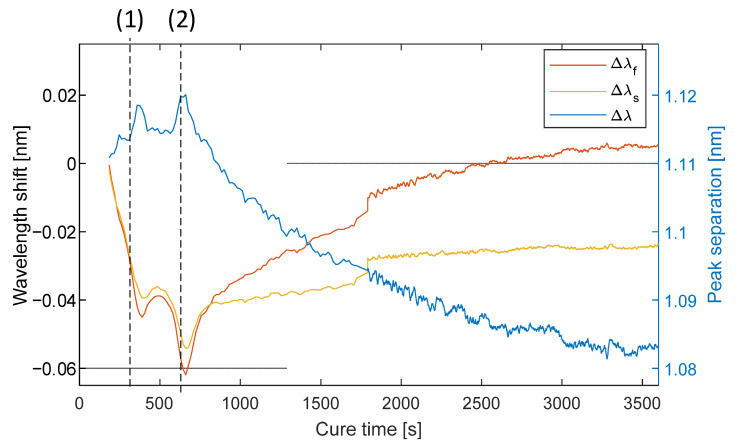
Bragg wavelengths and peak separation changes during the curing process; (1)—gelation time determined from data of the in-mold DC sensor, (2)—deadhesion of the plate from the mold.

The manufacturing process was repeated in the same conditions, but with the integration of HB FBG in the preform. After clamping of the injection hose, reflection optical spectra were collected. Figure 7 presents a relative change in the Bragg wavelength for both peaks (slow and fast) and peak separation of one sensor in the time domain:

When the resin is in the liquid state, the Bragg wavelengths of both peaks of FBG move parallelly, showing the decreasing trend. Around the gelation time (point (1) in Figure 7, 350 s), one can see the change of trend, which could be caused by the gelation of the resin. Afterwards, peaks are no longer moving parallelly and peak separation changes. Beginning at 550 s time, a trend is observed in decreasing peak separation, which is caused by the out-of-plane shrinking of the material. Temperature variations do not influence the peak separation; thus, the trend is not altered by the slight temperature variations caused by the heating system.

The trend in peak separation is interrupted at the 700 s time mark, probably caused by the detachment of the composite plate from the mold. In paper [6] regular FBGs measured strain variations in the stage between gel point and vitrification, which were attributed to the deadhesion of the plate from the mold. In-mold pressure sensors confirm the possibility of plate detachment in this stage of the process.

**Figure 8:**
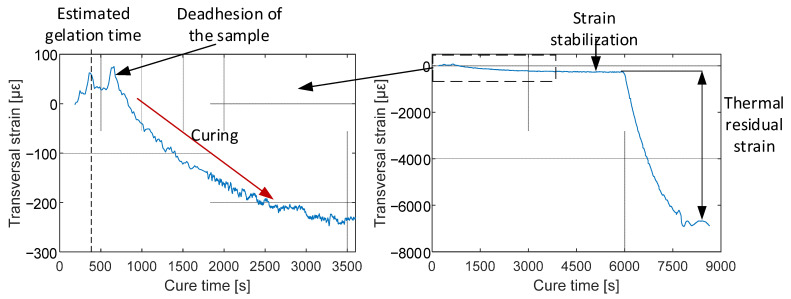
Through-thickness strain during manufacturing.

From mechanical testing of samples made out of this composite, a coefficient of peak separation changes to transversal strain was measured to be 0.12 pm/με [29], which was used to calculate transversal strain during the process; this coefficient is only an approximation, because strain transfer from composite to optical fiber varies during the manufacturing process. Calculated strain variation is presented in Figure 8.

Transversal strain after the curing process (chemical residual strain) was estimated to be −250 με; it stabilized around 2800 s, which is comparable to the curing time of this resin [33]. After 6000 s, mold’s heating was turned off and plate was cooled inside it. After complete cooling of a plate, the final transversal residual strain was −7000 με.

**Figure 9:**
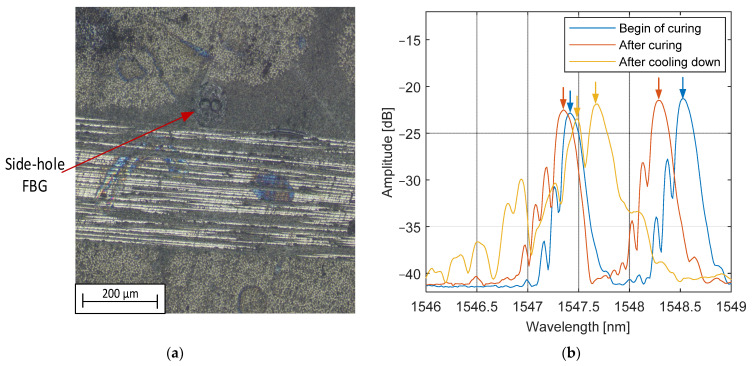
(**a**) The composite cross-section in the vicinity of the FBG sensor; (**b**) Optical spectrum of HB FBG before and after curing, and after cooling down.

The cross-section of the composite in the vicinity of the FBG sensor is shown in Figure 9a; it shows the angle orientation is the same as intended during the integration process. No voids were observed in the vicinity of the optical fiber.

The spectra of the FBG acquired (i) after injection, i.e., the start of the curing stage, (ii) after curing and (iii) after complete cooling down is shown in Figure 9b. Between the begin of cure and the end, only peak separation changed due to the transversal strain build-up. Cooling down causes further peak separation reduction, due to the difference in the coefficients of the expansion of the constitutive materials. Although the shape of the spectrum slightly degraded after cooling down, measurement of peak separation was still possible.

No significant splitting of peaks or spectra degradation was observed along the whole process; it means that strain was uniform on the length of the fiber, so no microbending or strain gradients were observed. The transversal strain however caused a significant decrease in the peak separation, which limits the range of strain sensing in the out-of-plane compression.

Since FBG functioned well, their integration method, and also manufacturing process did not compromise the operation of the FBG and they can be used for further Structural Health Monitoring, especially if robust peak detection methods are used [34].

## 5. Conclusions

In this paper, the usage of FBG sensors inscribed in regular single-mode and highly birefringent side-hole fiber was used to monitor the RTM process. The in-mold DC sensor was used as a reference gel point detection method. The determined gel point was compared with the results of monitoring with optical sensors. For the temperature compensation of FBG strain measurements, only in-mold sensors were used.

The following conclusions can be drawn:

1. Regular FBG sensors can be used to estimate the gelation point during the RTM process. However, chemical residual strain build-up in the plane of the composite is slow, so it is hard to determine precise boundaries for the gelation point. Moreover, slight changes in the temperature significantly influence the measurement; it seems that the use of regular FBG in the RTM process requires integrated temperature sensors for good compensation.
2. The build-up of transversal residual strain build-up can be measured with HB FBG sensors and is more distinct than the trend observed with regular FBG sensors. Peak separation sensitivity to temperature is low. For this reason, transversal strain measurement with FBG inscribed in side-hole elliptical core fiber doesn’t require another integrated sensor for temperature compensation
3. The advantage of HB FBG over FBG inscribed in regular FBG sensors was previously proposed in papers [20,27] for different manufacturing processes; this observation was also confirmed in this work.

## Figures and Tables

**Table 1:** Uses of FBG sensors to monitor composite manufacturing processes.

Used Type of FBG	Material	Manufacturing Process	Detected Strain	Goal of Study	Ref.
SM FBG	Neat epoxy resin	Curing in the oven temperature	Axial	Detection of neat resin cure process stages with FBG sensors and thermocouples	[17]
SM FBG	Flax-fiber twill with bio-epoxy	Resin Transfer Molding	Axial	Increasing of repeatability of process monitoring	[4]
SM FBG	Biaxial GF reinforcement, epoxy resin	Resin Transfer Molding	Axial	Monitoring of RTM process stages (flow front, curing)	[5]
SM FBG	GF mat, epoxy resin	Resin Transfer Molding	Axial	Detection of gel point and residual strain	[6]
SM FBG (small and regular diameter)	Unidirectional CF, epoxy resin	Vacuum infusion	Axial	Comparison of performance of FBG inscribed in 150 µm and 80 µm single-mode fibers	[19]
SM FBG	Unidirectional CF prepregs	Autoclave curing	Transversal, axial	Measurement of direction-dependent shrinkage with FBGs in plane and inserted transversally to the surface of prepreg stack	[21]
HB FBG (bow-tie fiber) SM FBG	CF placed by dry-fiber placement method	Infusion and autoclave curing,	Transversal, axial	Measurement of transversal and axial residual strain during the curing	[22]
HB FBG (bow-tie fiber)	Unidirectional GF prepregs	Open-air curing	Transversal	Detection of gel point and of cure with HB FBG	[27]
HB FBG (microstructured fiber)	Symmetric CF laminate, epoxy resin	Autoclave curing	Transversal	Measurement of final transversal-strain measurement after the curing process	[28]

## Data Availability

The data supporting this paper are available upon request by contact with the corresponding author.
